# The impact of nerve management on the risk for persistent postoperative pain one year after open anterior mesh inguinal hernia repair

**DOI:** 10.1007/s10029-025-03510-9

**Published:** 2026-01-20

**Authors:** Linn Westin, G Sandblom, U Gunnarsson, U Dahlstrand

**Affiliations:** 1 Department of Trauma and Reparative Medicine, Karolinska University Hospital, Stockholm, 141 86 Sweden; 2 Karolinska Institutet, Department of Clinical Science, Intervention and Technology – CLINTEC, and Karolinska Universitetssjukhuset, Stockholm, Sweden; 3 Department of Clinical Science and Education Södersjukhuset, Karolinska Institutet, Stockholm, Sweden; 4 Department of Surgery, Södersjukhuset, Södersjukhuset, Sweden; 5 Department of Surgical and Perioperative Science, division of Surgery, Umeå University, Umeå, Sweden; 6 Department of Surgery, Enköping Hospital, Enköping, Sweden

**Keywords:** Pain, Nerves, Open repair, Register

## Abstract

**Purpose:**

Persistent postoperative pain is a major challenge in inguinal hernia surgery. However, the impact of intraoperative nerve management on postoperative pain is poorly understood. The aim was to evaluate how management of the three inguinal nerves during anterior mesh repair of inguinal hernia affects the risk for persistent postoperative pain.

**Methods:**

Cohort study based on data from the Swedish Hernia Register (SHR) concerning management of the three inguinal nerves. Adult patients with an open anterior mesh repair between 2012 and 2017 and who had responded to a patient-reported outcome measure (PROM) questionnaire one year after surgery were included in the study.

**Results:**

Out of eligible patients, 34,115 (69%) responded to the PROM questionnaire. Of these, 25.9% reported pain that could not be ignored and 15.7% reported pain interfering with daily activities one year after surgery. Identifying and/or preserving any of the three groin nerves was not seen to have a significant impact on the risk for persistent groin pain in multivariable ordinal regression analysis adjusted for type of anaesthesia, gender, age and emergency surgery.

**Conclusion:**

In a setting where the nerves are handled according to the surgeon’s intraoperative judgement focusing on identifying and/or preserving the nerves, there was no association between intraoperative management of the three inguinal nerves and the risk for persistent postoperative pain one year after surgery. While careful tissue handling is crucial to the avoidance of postoperative pain, pragmatic nerve resection did not increase the risk for persistent pain one year after surgery.

## Introduction

Inguinal hernia is a benign yet frequent condition which is only cured by surgery. In Sweden alone around 16,000 hernia repairs are performed each year, and in the United States of America that figure is 800,000, making it the most common surgical procedure in that nation [1]. As for any surgical intervention, prevention of complications after inguinal hernia surgery is crucial to the health-related quality-of-life of these patients. Identifying risk factors for complications is key to developing the means to reduce risk and improve outcome after inguinal hernia repair.

Since the introduction of synthetic mesh in hernia surgery, the risk for recurrence has been substantially reduced [2, 3]. If not contraindicated, the use of mesh is now considered standard care in inguinal hernia surgery. Mesh was used in 99.5% of all inguinal hernia repairs performed in Sweden during 2023 [4]. Until recently, recurrence was considered the most important surgical outcome. However, more commonly occurring complications have now taken priority in our efforts to improve quality, the most notable being persistent pain. Many patients undergoing inguinal hernia surgery are of working age and have high demands regarding physical activity. Having to avoid motion-related pain may have a substantial impact on their role in society and their physical abilities.

The underlying cause of persistent pain after inguinal hernia surgery is not well described. Several factors have been linked to increased risk for pain including young age, female gender, type of anaesthesia, emergency surgery, and intensity of pain prior to surgery [4–7]. Persistent pain rates of up to 30% have been reported [4, 7, 8]. It is probable that both neuropathic and nociceptive mechanisms are involved in persistent pain. Neuropathic pain was cited as the cause of persistent pain in 68% of patients after inguinal hernia repair [9, 10].

There are three major sensory nerves in the groin that traverse the surgical field during open anterior inguinal hernia repair: the ilioinguinal nerve, the iliohypogastric nerve, and the genital branch of the genitofemoral nerve [10]. The association between handling of these three nerves and the risk for postoperative pain has not been widely studied, though studies on prophylactic resection of these nerves have been published [11, 12].

Identification of the nerves, and pragmatic resection of these in the case of injury or risk for injury is recommended [13], however evidence in this area of research is not strong. To our knowledge there are no randomised trials on the subject, and only a few cohort studies [14–16]. Negative side-effects of this approach are limited, and thus the concept of nerve recognition and pragmatic resection has been adopted in Sweden and taught in surgical education over the last decade.

The objective of this study was to evaluate the prevalence of persistent pain one year after surgery and comparison of this with intraoperative management of the ilioinguinal, iliohypogastric and genitofemoral nerves. Our null hypothesis was that there is a lower risk for persistent pain in the operated groin if these nerves are identified and spared during hernia repair.

## Methods

### Study design and participants

The study was designed as a cohort study based on data from the Swedish Hernia Register (SHR). Ninety operating units participate in SHR representing 95% of hospitals and clinics performing inguinal hernia repair in Sweden and it has been shown to have high validity [11]. Patients undergoing inguinal hernia surgery at participating clinics are informed that research is conducted on data in the SHR and that they can refuse any registration of their data. After approval by the Swedish Ethics Review Authority (dnr: 2019–02487), data were retrieved from the SHR.

Handling of the three nerves during open anterior repair has been registered in SHR since 2012 [11]. The categories registered are ‘identification and spared’, ‘identification and resected’, or ‘not identified’ for each of the ilioinguinal, the iliohypogastric and the genital branch of the genitofemoral nerves. A patient-reported outcome measure (PROM) questionnaire was sent one year after surgery to all patients who had a repair between 1 st September 2012 and 30th August 2017.

The PROM questionnaire had one section assigned to general health and a second section that focused on items related to pain in the operated area. All items have been adapted and modified to fit the patient population undergoing hernia repair. The items related to pain were derived from the Inguinal Pain Questionnaire [17]. Intensity of pain was reported on an ordinal scale ranging from 1 to 7 where 1 represents no pain and 7 pain necessitating immediate contact with healthcare.

**Fig. 1:**
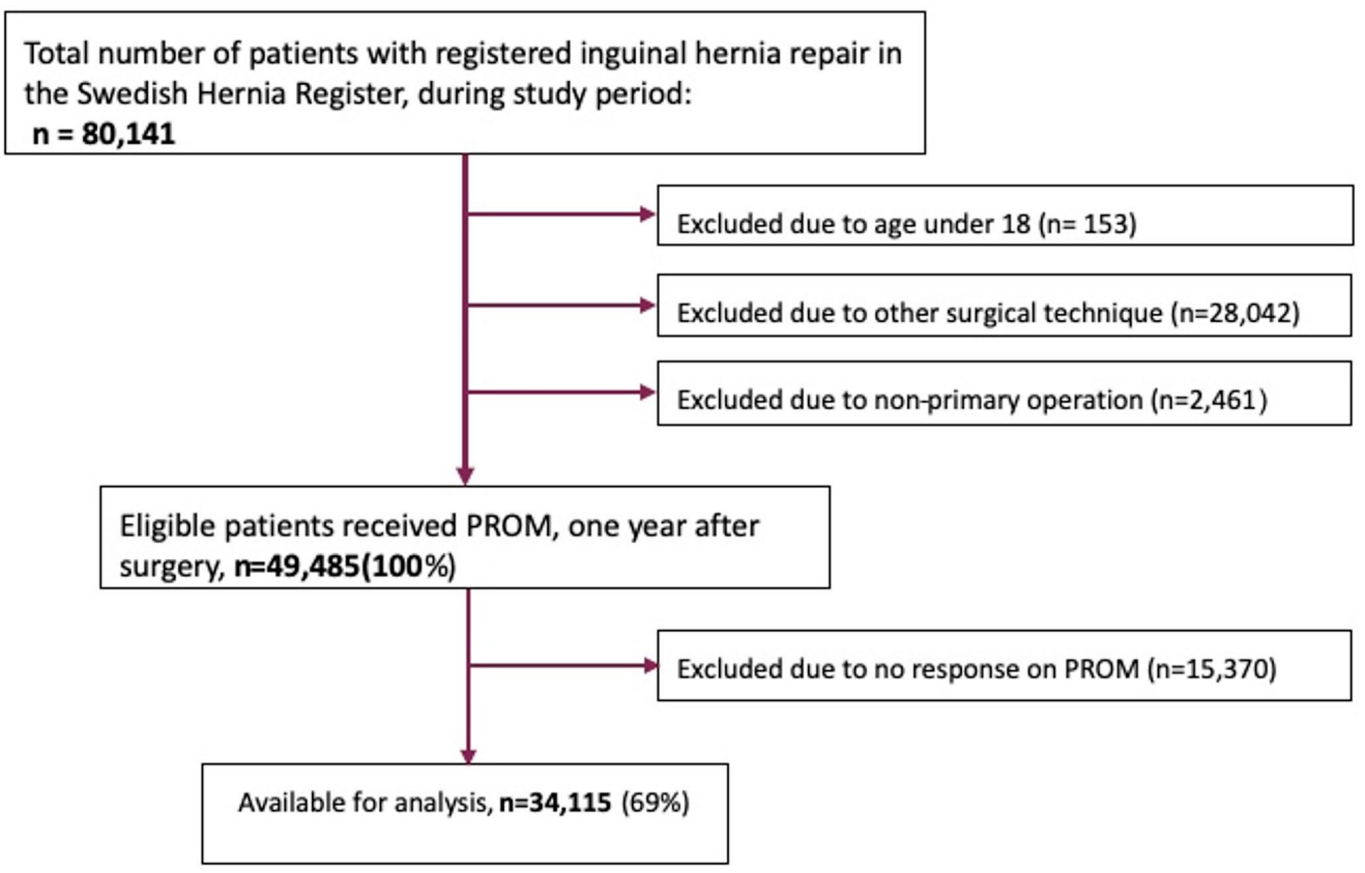
Flow diagram of identification, exclusion, and inclusion of study subjects

Data from the SHR were requested for procedures carried out from the beginning of 2012 until the end of 2017. Management of the individual nerves and registration began in 2012. Inclusion criteria were age ≥ 18 years at time of surgery, primary open anterior mesh repair (not including plug repairs), and completed PROM questionnaire. A flow-chart of the inclusion process is presented in Fig. 1.

### Outcomes

The primary outcome measure was pain defined as “most intense pain perceived the week before” one year after surgery as rated in the PROM questionnaire on a seven-point ordinal scale.

Statistical analyses:

Statistical analyses were performed using Stata/IC version 15.0 (StataCorp, College Station, TX, USA). Ordinal logistic regression for the outcome, pain, was applied in two separate analyses. In the main analysis, handling of each of the three nerves were included in the model as independent variables, with four previously documented factors shown to have an impact on postoperative pain i.e., age, type of anaesthesia, emergency surgery, and gender, as covariates. The second model considered the overall management of nerves in three categories: ‘all identified and spared’, ‘none identified’, or 'all other combinations' for each of the ilioinguinal, the iliohypogastric and the genital branch of the genitofemoral nerves. The first category acted as reference group. All other covariates were the same as in the first analysis.

## Results

The SHR included 80,141 inguinal hernia repairs carried out during the study period, 49,485 of which were primary open anterior mesh repairs on patients 18-years-of-age or above. All patients received the PROM questionnaire and 34,115 (69%) patients answered.

Patient characteristics of the group answering the PROM compared to the non-responders are presented in Table 1. In this population of patients that underwent a primary open anterior repair, the majority were men (> 96%) and the median age was 67 years for responders and 61.5 years for non-responders.

**Table 1:** Patient characteristics of the group responding to the PROM versus non-responders among patients with an open anterior mesh repair

Patient Characteristics	Responders (n=34,115)	Non-responders (n=15,388)
Age, median (IQR)	67 (39.1-83.0)	61.5 (28.7-84.9)
Male, n (%)	34,843 (97.5)	15,671 (96.6)
BMI, median (IQR)	25.0 (20.9-30.4)	25.1 (20.5-31.2)
ASA classification, n (%)		
I	14,127 (41.4)	6,865 (44.6)
II	15,613 (45.8)	6,062 (39.4)
III	4,252 (12.5)	2,275(14.8)
IV	122 (0.4)	184 (1.2)
Hernia anatomy, n(%)		
Lateral	19,439 (57.0)	8,821 (57.3)
Medial	11,314 (33.2)	5,081 (33.0)
Femoral	114 (0.3)	73 (0.5)
Combined	2,990 (8.8)	1,173 (7.6)

The pattern of nerve recognition varied between the three nerves. The ilioinguinal nerve was identified in 71.5% of cases, of which it was spared in 59.7% and resected in 11.8%. The iliohypogastric nerve was identified in 52.6% of cases, spared in 43.5% and resected in 9.1%. The genital branch of the genitofemoral nerve went un-identified in 78.2%, was identified and spared in 17.9% and identified and resected in 3.9% of the cases. The pattern of nerve recognition and treatment was very similar among non-respondents.

The prevalence of pain (discomfort that could not be ignored, score 2 or more on the 7-point scale) was 25,9%. For severe pain (pain that interfered with daily activities, score 4 or more on the 7-point scale) the prevalence was 15.7%. In the univariable ordinal regression analysis, there was no significant association between pain and management of any of the nerves.

Results from the main multivariable ordinal logistic regression regarding pain one year after surgery are presented in Table 2. Persistent pain was not associated with non-identification of the ilioinguinal nerve (OR 1.00, 95% CI 0.95–1.06), or with identification and resection (OR 0.98, 95% CI 0.92–1.05) when compared to identification and spared as reference. Similar results were seen with the iliohypogastric nerve (non-identification OR 1.04, CI 0.99–1.09 and identification + resection OR 0.99, CI 0.92–1.07, respectively) and with the genitofemoral nerve (non- identification OR 0.98, CI 0.93–1.03, and identification + resection OR 0.96, CI 0.86–1.08, respectively). Female gender and age under 61 years were significant risk factors for persistent pain and for the degree of pain (*p* < 0.001). It was also seen that patients with a BMI over 25 kg/m^2^ had an increased risk for pain (*p* < 0.001) as did patients with ASA classification II or III-IV (*p* < 0.001 and *p* = 0.003).

**Table 2.** Uni- and multivariable ordinal logistic regression on level of pain in the operated groin one year after surgery. Study population of 34,115 patients with anterior mesh repair for groin hernia 2012 - 2017

Variable		Univariable			Multivariable	
	Odds ratio	p-value	95% Conf. Interval	Odds Ratio	p-value	95% Conf. Interval
Ilioinguinal nerve, identified and spared (ref)						
Not identified	1.02	0.535	0.97–1.06	1.00	0.878	0.95–1.06
Identified and resected	0.97	0.339	0.91–1.03	0.98	0.522	0.92–1.05
Iliohypogastric nerve, identified and spared (ref)						
Not identified	1.04	0.103	0.99–1.08	1.04	0.089	0.99–1.09
Identified and resected	0.99	0.739	0.92–1.06	0.99	0.935	0.92–1.07
Genital branch of genitofemoral nerve, identified and spared (ref)						
Not identified	0.99	0.725	0.94–1.04	0.98	0.432	0.93–1.03
Identified and resected	0.96	0.450	0.86–1.07	0.96	0.486	0.86–1.08
Male gender	0.73	< 0.001	0.64–0.82	0.74	< 0.001	0.65–0.84
General anaesthesia (ref)						
Epidural or spinal anaesthesia	1.02	0.750	0.92–1.12	1.11	0.038	1.01–1.23
Local anaesthesia	0.92	0.002	0.87–0.97	0.97	0.234	0.92–1.02
Emergency surgery	1.07	0.234	0.96–1.19	1.08	0.178	0.96–1.22
Age 61 and above (ref)						
18–40	1.75	< 0.001	1.61–1.90	1.88	< 0.001	1.72–2.05
41–60	1.43	< 0.001	1.36–1.49	1.49	< 0.001	1.42–1.56
ASA I (ref)						
ASA II	1.14	< 0.001	1.07–1.22	1.06	< 0.001	1.02–1.18
ASA III & IV	1.05	0.139	0.98–1.12	1.30	< 0.001	1.21–1.39
BMI 20–25 (ref)						
< 20	1.06	0.057	0.99–1.13	1.05	0.082	0.99–1.12
> 25	1.21	< 0.001	1.16–1.29	1.19	<0.001	1.14–1.24

In the second multivariable analysis, all three nerves were identified and spared in 3,271 cases, whereas in 5,974 cases, no nerve was identified. Identification and sparing of all nerves had no significant impact on the outcome of pain. However, the risk for pain one year after surgery was higher in cases where no nerve had been identified (OR 1.08, CI 1.02–1.14, *p* = 0.007).

## Discussion

Management of the three nerves that traverse the surgical field during open anterior hernia repair was not associated with risk for persistent pain one year after surgery in this national quality register population-based study. This is in accordance with findings by Bischoff et al. in a small clinical study including 224 patients with unilateral primary hernia undergoing open anterior mesh repair [18]. There was, however, a higher risk for persistent pain in cases where no nerve was identified. The present study has a larger cohort covering many clinics. The material represents routine hernia surgery in Sweden and is therefore generalisable. In an observational study, Magnusson et al. looked at the effect of nerve management on sensory disorder and persistent pain in 157 patients undergoing hernia repair [19]. As in the present study, no association was seen between persistent pain and nerve management.

In accordance with previous studies, we found that age and gender had a significant effect on the risk for persistent postoperative pain [19, 20]. However, no significant association was seen between emergency surgery and pain reported one year after surgery.

The prevalence of pain in the present study was comparable to those in previous reports [4] which strengthens the external validity and clinical relevance of our findings. However, the actual prevalence of persistent pain may vary due to selection bias of patients responding to the questionnaire. It is possible, for instance, that those not experiencing discomfort after surgery are less inclined to spend their time answering a questionnaire one year after surgery as described by Lundström et al. [17]. It has also been shown that patients who have undergone endoscopic repair have less pain at one year and later [20, 21]. Patients who underwent minimally invasive procedures were excluded from this study since dissection from the posterior approach generally does not interfere with the three nerves investigated. Furthermore, nerve management is not registered in the SHR for these patients.

The internal validity of this study depends on the accuracy of reporting of management of the nerves during surgery. In the Swedish Hernia Register, nerve management has been reported as a separate variable since 2012, though this is a procedural report not a quality outcome measure. As shown by Axman et al., the validity of the register is high, and 98% of items are accurately registered [11]. Nevertheless, it cannot entirely be ruled out that there were erroneous or inaccurate statements in the patient records that would not be corroborated in an external review. A limitation of the study is the lack of control of other factors which may affect the risk for chronic postoperative pain, such as mesh material and fixation or whether the deep inguinal ring was narrowed prior to mesh placement for example. The SHR covers 95% of groin hernia repairs in Sweden and is structured in a standardised way. It thus covers virtually the entire Swedish hernia population, which limits the risk of selection bias.

During dissection and repair of the hernia and when placing the mesh, there is a chance that a nerve is injured or entrapped in the mesh repair. When this occurs, the decision whether to dissect around the nerve or resect it must be made. It must be stressed that while careful dissection with attention to identification of nerves and other tissues is essential for successful hernia repair, it is not recommended to perform extensive dissection in order to identify nerves that are not encountered. This study found a higher risk for persistent pain after procedures where no nerve had been identified. Several reasons could explain this such as non-identification of the nerves indicating difficult dissection of a complex groin hernia or the surgeon having limited understanding of the surgical anatomy. When nerves are not identified they are at greater risk of entrapment in sutures fixing the mesh as well as of injuries and transection. To minimize the risk for entrapment attention to detail in orientation of medial sutures, not tying them down tightly and avoiding placement in areas where an intramuscular course is to be expected is warranted. As several of the known risk factors for chronic postoperative pain are non-modifiable, such as age or gender, they can only be mitigated by choosing suitable techniques and executing them as well as possible. Since it is now mandatory in the SHR to register how each of the three nerves was managed, there is greater focus on identification of the nerves and their dissection and management. The practically 100% participation of surgical units in SHR has led to very few surgeons not reflecting on these aspects when performing open groin hernia repair. As described previously, there can be adequate reasons to resect the nerve as it traverses the operating field and may interfere with fixation of the mesh or risk being compromised by the mesh after repair. Both dissection injuries to nerves and trauma from mesh edges in direct contact with a nerve can result in neuropathic pain. Smaller controlled studies have shown less pain reported after consistently resecting nerves that were at risk during surgery [12, 13]. The approach of pragmatic resection is recommended in the “International guidelines for groin hernia management” issued by the HerniaSurge Group, and this has been embraced in Sweden [13]. The present study was an observational study on routine hernia repairs in a population-based cohort i.e., how open hernia surgery is practiced in the Swedish community at large. In each case, the nerves were managed as deemed best by the surgeon. While we do not know the exact reasoning behind the surgeon’s choice, the strength of the study is the large number of patients included and the high validity of the SHR, contributing to the generalisability of the results.

In conclusion, data from the Swedish Hernia Register support continued pragmatic management of inguinal nerves during open anterior repair, where focus is on nerve identification.

## References

[1] Rutkow IM (2003) Demographic and socioeconomic aspects of hernia repair in the United States in 2003. Surg Clin North Am 83:1045–105114533902 10.1016/S0039-6109(03)00132-4

[2] EU Hernia Trialists Collaboration (2000) Mesh compared with non-mesh methods of open groin hernia repair: systematic review of randomized controlled trials. Br J Surg 87:854–85910931018 10.1046/j.1365-2168.2000.01539.x

[3] HerniaSurge Group (2018) International guidelines for groin hernia management. Hernia 22:1–165 10.1007/s10029-017-1668-x PMC5809582 29330835

[4] Swedish Hernia Register (2023) Annual report 2023. Available at: https://www.svensktbrackregister.se/images/Årsrapporter/Ny_årsrapport_23.pdf. Accessed Nov 2024

[5] Nienhuijs S, Staal E, Strobbe L, Rosman C, Groenewoud H, Bleichrodt R (2007) Persistent pain after mesh repair of inguinal hernia: a systematic review. Am J Surg 194:394–40017693290 10.1016/j.amjsurg.2007.02.012

[6] Prakash D, Heskin L, Doherty S, Galvin R (2017) Local anaesthesia versus spinal anaesthesia in inguinal hernia repair: a systematic review and meta-analysis. Surgeon 15:47–5726895656 10.1016/j.surge.2016.01.001

[7] Nordin P, Zetterström H, Gunnarsson U, Nilsson E (2003) Local, regional, or general anaesthesia in groin hernia repair: multicentre randomised trial. Lancet 362:853–85813678971 10.1016/S0140-6736(03)14339-5

[8] Franneby U, Sandblom G, Nordin P, Nyrén O, Gunnarsson U (2006) Risk factors for persistent pain after hernia surgery. Ann Surg 244:212–21916858183 10.1097/01.sla.0000218081.53940.01PMC1602172

[9] Callesen T, Bech K, Kehlet H (1999) Prospective study of persistent pain after groin hernia repair. Br J Surg 86:1528–153110594500 10.1046/j.1365-2168.1999.01320.x

[10] Kalliomäki ML, Sandblom G, Gunnarsson U, Gordh T (2009) Persistent pain after groin hernia surgery: a qualitative analysis of pain and its consequences for quality of life. Acta Anaesthesiol Scand 53:236–24619094175 10.1111/j.1399-6576.2008.01840.x

[11] Axman E, Nordin P, Modin M, de la Croix H (2021) Assessing the validity and cover rate of the National Swedish hernia register. Clin Epidemiol 13:1129–113434938123 10.2147/CLEP.S335765PMC8687441

[12] Hsu W, Chen CS, Lee HC, Liang HH, Kuo LJ, Wei PL, Tam KW (2012) Preservation versus division of ilioinguinal nerve on open mesh repair of inguinal hernia: a meta-analysis of randomized controlled trials. World J Surg 36:2311–231922644622 10.1007/s00268-012-1657-2

[13] Charalambous MP, Charalambous CP (2018) Incidence of persistent groin pain following open mesh inguinal hernia repair, and effect of elective division of the ilioinguinal nerve: meta-analysis of randomized controlled trials. Hernia 22:401–40929550948 10.1007/s10029-018-1753-9

[14] Alfieri S, Amid PK, Campanelli G, Izard G, Kehlet H, Wijsmuller AR et al (2011) International guidelines for prevention and management of post-operative persistent pain following inguinal hernia surgery. Hernia 15:239–24921365287 10.1007/s10029-011-0798-9

[15] Bartlett DC, Porter C, Kingsnorth AN (2007) A pragmatic approach to cutaneous nerve division during open inguinal hernia repair. Hernia 11:243–24617541702 10.1007/s10029-007-0209-4

[16] Reinpold W, Nehls J, Eggert A (2011) Nerve management and persistent pain after open inguinal hernia repair: a prospective two phase study. Ann Surg 254:163–16821562403 10.1097/SLA.0b013e31821d4a2d

[17] Fränneby U, Gunnarsson U, Andersson M, Heuman R, Nordin P, Nyrén O, Sandblom G (2008) Validation of an inguinal pain questionnaire for assessment of persistent pain after groin hernia repair. Br J Surg 95:488–49318161900 10.1002/bjs.6014

[18] Bischoff JM, Aasvang EK, Kehlet H, Werner MU (2012) Does nerve identification during open inguinal herniorrhaphy reduce the risk of nerve damage and persistent pain? Hernia 16:573–57722782363 10.1007/s10029-012-0946-x

[19] Magnusson N, Hedberg M, Österberg J, Sandblom G (2010) Sensory disturbances and neuropathic pain after inguinal hernia surgery. Scand J Pain 1:108–11129913947 10.1016/j.sjpain.2010.01.004

[20] Reinpold W (2017) Risk factors of persistent pain after inguinal hernia repair: a systematic review. Innov Surg Sci 2:61–6831579738 10.1515/iss-2017-0017PMC6754000

[21] Westin L, Wollert S, Ljungdahl M, Sandblom G, Gunnarsson U, Dahlstrand U (2016) Less pain 1 year after total extra-peritoneal repair compared with Lichtenstein using local anesthesia: data from a randomized controlled clinical trial. Ann Surg 263:240–24326079901 10.1097/SLA.0000000000001289

